# The potential of anti-malarial compounds derived from African medicinal plants, part I: a pharmacological evaluation of alkaloids and terpenoids

**DOI:** 10.1186/1475-2875-12-449

**Published:** 2013-12-13

**Authors:** Pascal Amoa Onguéné, Fidele Ntie-Kang, Lydia Likowo Lifongo, Jean Claude Ndom, Wolfgang Sippl, Luc Meva’a Mbaze

**Affiliations:** 1 Department of Chemistry, Faculty of Science, University of Douala, PO Box 24157, Douala, Cameroon; 2 Chemical and Bioactivity Information Centre, Department of Chemistry, Faculty of Science, University of Buea, PO Box 63, Buea, Cameroon; 3 Department of Pharmaceutical Sciences, Martin-Luther University of Halle-Wittenberg, Wolfgang-Langenbeck Str. 4, Halle, Saale 06120, Germany

**Keywords:** Africa, Malaria, Medicinal plants, Natural products, Traditional medicine

## Abstract

Traditional medicine caters for about 80% of the health care needs of many rural populations around the world, especially in developing countries. In addition, plant-derived compounds have played key roles in drug discovery. Malaria is currently a public health concern in many countries in the world due to factors such as chemotherapy faced by resistance, poor hygienic conditions, poorly managed vector control programmes and no approved vaccines. In this review, an attempt has been made to assess the value of African medicinal plants for drug discovery by discussing the anti-malarial virtue of the derived phytochemicals that have been tested by *in vitro* and *in vivo* assays. This survey was focused on pure compounds derived from African flora which have exhibited anti-malarial properties with activities ranging from “very active” to “weakly active”. However, only the compounds which showed anti-malarial activities from “very active” to “moderately active” are discussed in this review. The activity of 278 compounds, mainly alkaloids, terpenoids, flavonoids, coumarines, phenolics, polyacetylenes, xanthones, quinones, steroids, and lignans have been discussed. The first part of this review series covers the activity of 171 compounds belonging to the alkaloid and terpenoid classes. Data available in the literature indicated that African flora hold an enormous potential for the development of phytomedicines for malaria.

## Background

**Figure 1:**
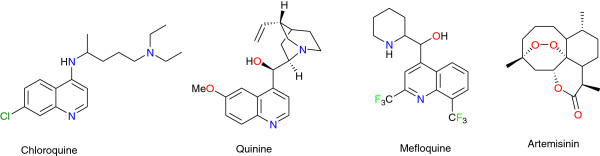
Some plant-derived anti-malarial drugs.

Malaria is an infectious disease with ravaging effects in the world. The World Health Organization (WHO) has published statistics which reveal that half the world’s population is at risk of malaria and that one to two million annual deaths can be attributed to malaria alone [1,2]. Four protozoan species of the genus *Plasmodium* (*Plasmodium falciparum*, *Plasmodium malariae*, *Plasmodium ovale*, and *Plasmodium vivax*) are responsible for this infection, although the majority of fatal cases are caused by *P. falciparum*[3]. Malaria has been treated with quinine, chloroquine, mefloquine, and artemisinin (Figure 1), among other drugs. However, the protozoans have developed resistance against many of the current treatment regimens [4]. In the quest to identify new anti-malarial chemotherapeutic agents, many research groups have resorted to plant sources [3,5,6]. This is because of the use of many of these plant materials in the treatment of malaria and fevers in African traditional medicine (ATM) [7]. There has been a general call for the use of natural products as drugs for malaria or as sources of inspiration for the development of novel anti-malarials [8-11] in order to possibly avoid problems related to drug resistance [12].

The African continent is very rich in floral biodiversity and its plant materials are endowed with natural products (NPs) with intriguing chemical structures and promising biological activities. Therefore, the next generation anti-malarials or the scaffolds necessary for their synthesis may be found in plants currently used in ATM [13,14]. It should also be mentioned that malaria mostly affects the populations of Africa, Asia and Latin Africa. Asia has offered artemisinin to humanity while Latin America has offered quinine. Many researchers are therefore of the opinion that it is Africa’s turn to offer a new anti-malarial drug to humanity. Why do we not yet find a (real) anti-malarial drug from Africa? This brings us to the need to have an overview of the anti-malarial/anti-plasmodial activity of compounds from bitter African plants (alkaloids and terpenoids). Several research groups in Africa have been involved in the bioassay-guided fractionation of plant extracts, leading to the isolation, purification and characterization of a significant number of NPs, some with remarkable anti-malarial activities. The literature survey reported in this work has led to the identification of several vast screening efforts of crude extracts derived from plants used in ATM, harvested from the following countries, just to mention a few: the Democratic Republic of Congo [15,16], Nigeria [17-19], Mozambique, Cape Verde, Guinea-Bissau, São Tomé and Príncipe and Angola [20], Mali and São Tomé and Príncipe [21], Madagascar [22-24], Congo [25], Benin [26], Burkina Faso [27], South Africa [28], Ivory Coast [29], West African countries [30], Tanzania [31], Kenya [32], and East African countries [33,34].

The potential of plant-derived NPs for anti-malarial drug discovery has been examined in a number of review papers [3,35-41]. Other review articles have concentrated on anti-malarials from specific countries/regions in Africa [19,20,42-46]. However, there has been no review offering coverage of promising anti-malarials from the entire African continent in the last ten years [13]. In this review series, the potential of plant-derived NPs that could be developed into drugs have been discussed, by giving an overview of the most pertinent *in vitro* and *in vivo* screening results reported in the literature.

### Promising anti-malarial alkaloids and terpenoids derived from African flora

#### Alkaloids

**Figure 2:**
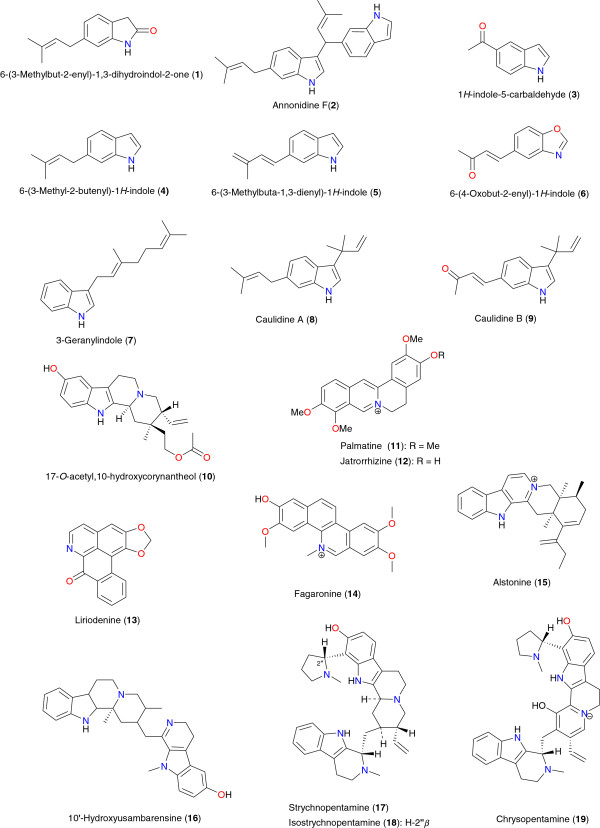
Anti-malarial indole alkaloids derived from the African flora.

**Figure 3:**
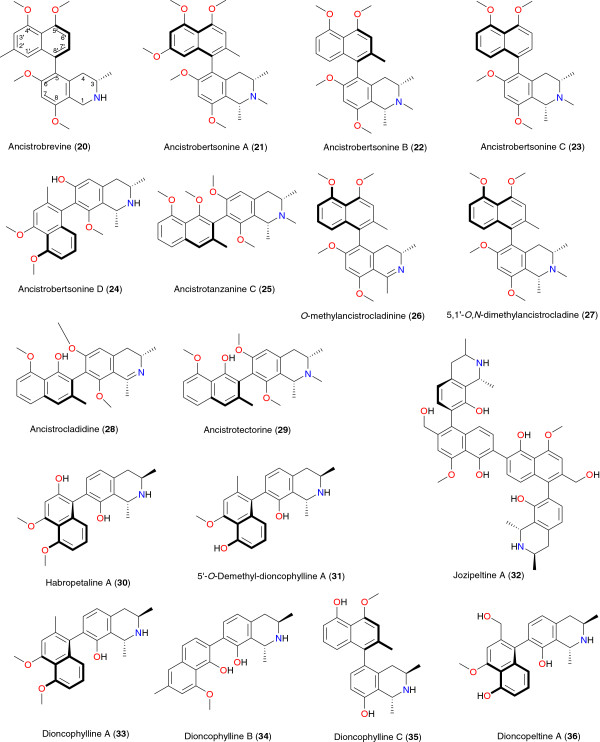
Naphthoisoquinolines with anti-plasmodial activity, derived from plants used in African traditional medicine.

**Figure 4:**
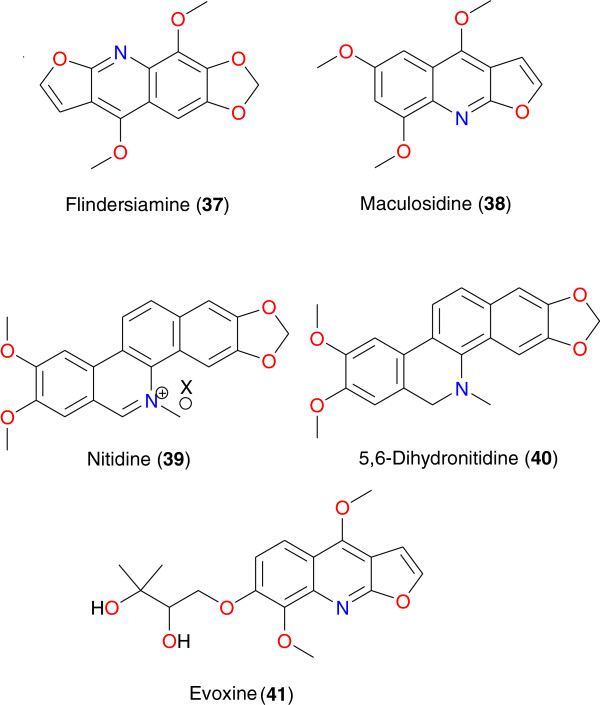
Promising anti-malarial furoquinolines from African medicinal plants.

**Figure 5:**
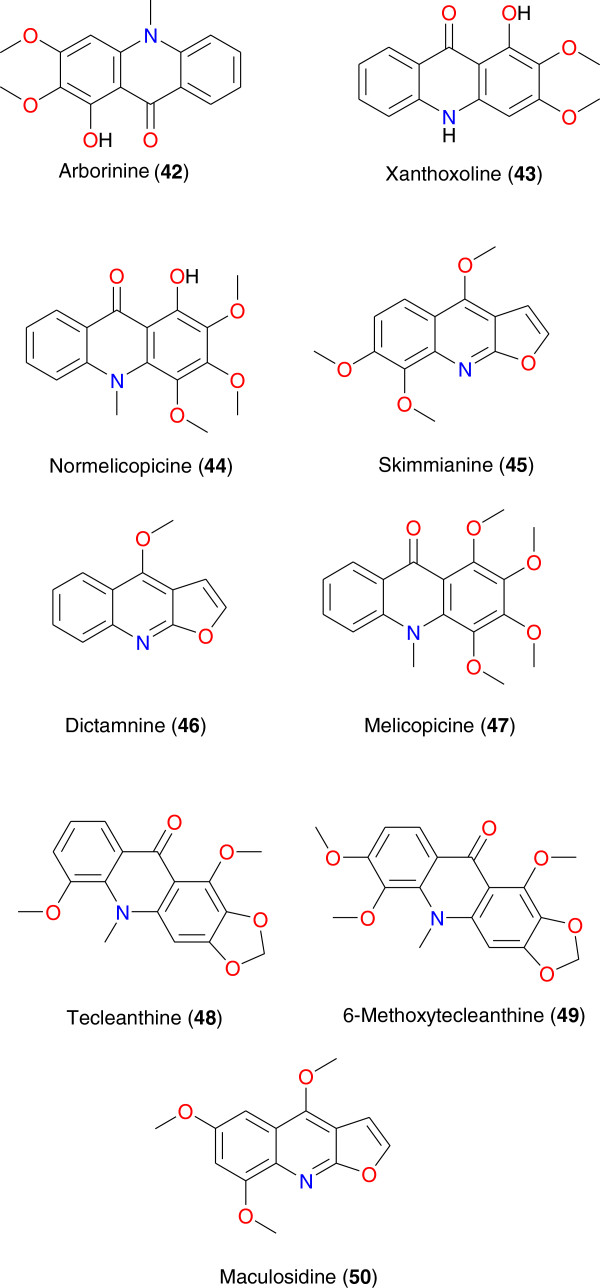
Promising anti-malarial acridones from African medicinal plants.

**Figure 6:**
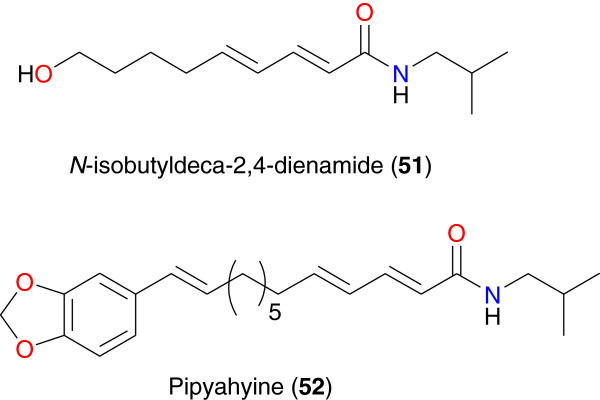
Some anti-malarial amides from African medicinal plants.

**Figure 7:**
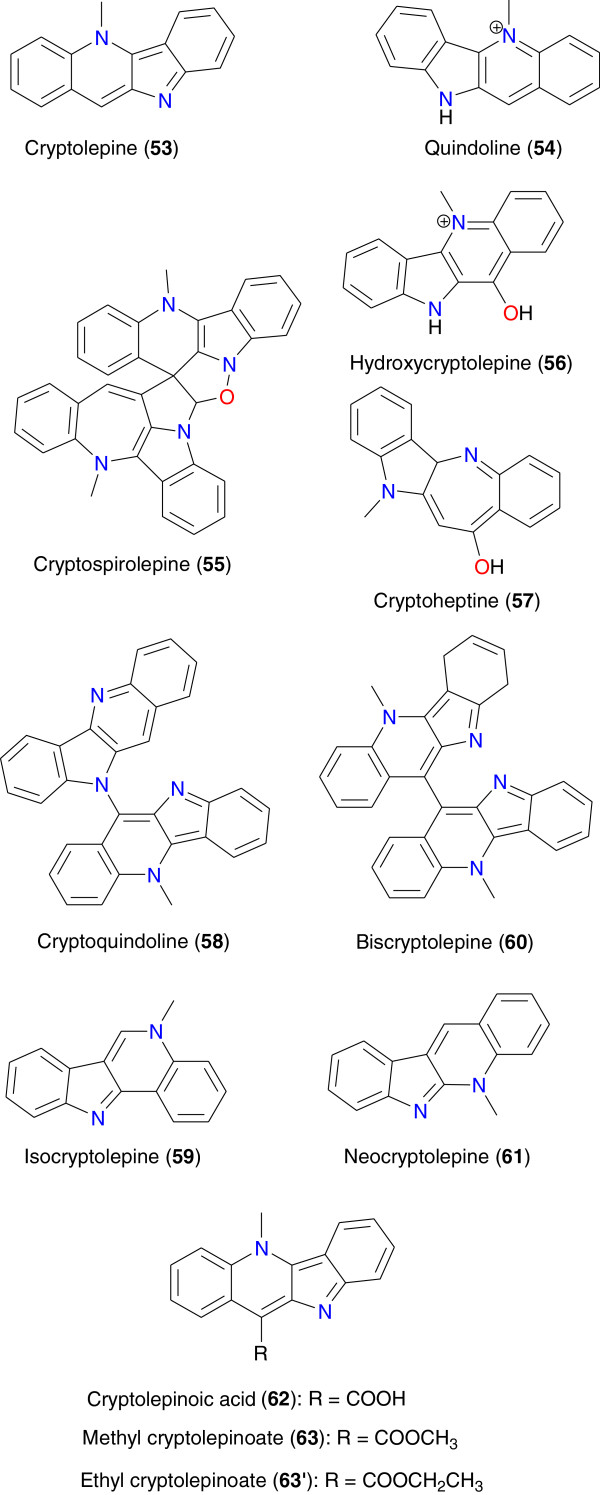
Anti-malarial cryptolepines from African medicinal plants.

Previous studies have shown that plant-derived alkaloids have a great potential for anti-malarial drug development [33-36,39,40,42-44]. Tables 1 and 2 summarize the most promising alkaloids derived from African medicinal plants with significant anti-malarial properties. The chemical structures are shown in Figures 2, 3, 4, 5, 6 and 7, according to the alkaloid subclasses.

**Table 1:** Summary of anti-malarial alkaloids derived from the African flora – indoles, naphthoisoquinolines and furoquinolines

**Compound subclass**	**Isolated metabolites**	**Plant species (Family)**	**Part of plant studied**	**Place of harvest (locality, country)**	**Author, reference**
Indole alkaloids	**1, 2, 3, 4, 5, 6** and **7**	*Monodora angolensis* (Annonaceae)	Stem and root bark	Kiwanda, Tanzania	Nkunya *et al.*[47]
	**8** and **9**	*Isolona cauliflora* (Annonaceae)	Stem and root bark, and flower stalks	Namikwe Island, Tanzania	Nkunya *et al.*[47]
	**10**	*Strychnos usambarensis* (Loganiaceae)	Leaves	Akagera National Park, Rwanda	Cao *et al.*[48]
	**11** and **12**	*Penianthus longifolius* (Menispermaceae)	Stem bark	Cameroon	Bidla *et al.*[49]
	**13**	*Glossocalyx brevipes* (Siparunaceae)	Leaves	Kumba, Cameroon	Mbah *et al*. [50]
	**14**	*Fagara zanthoxyloides* (Rutaceae)	Roots	Nigeria	Odebiyi *et al.*[51]
	**15**	*Picralima nitida* (Apocynaceae)	Fruits	Nnewi, Nigeria	Okunji *et al.*[52]
	**16**	*Strychnos usambarensis* (Loganiaceae)	Roots	Akagera National Park, Rwanda	Frédérich *et al.*[55]
	**17, 18** and **19**	*Strychnos usambarensis* (Loganiaceae)	Leaves	Akagera National Park, Rwanda	Frédérich *et al.*[56]
Naphthoisoquinolines	**20, 21, 22, 23** and **24**	*Ancistrocladus robertsoniorum* (Acistrocladaceae)	Stems and leaves	Buda Mafisini Forest, Kenya	Bringmann *et al.*[57]
	**25, 26, 27, 28** and **29**	*Ancistrocladus tanzaniensis* (Acistrocladaceae)	Leaves	Uzungwa Mountains, Tanzania	Bringmann *et al.*[58]
	**30**	*Triphyophyllum peltatum* (Dioncophyllaceae)	Roots	Parc de Taï, West Ivory Coast	Bringmann *et al.*[59]
	**31**	*Triphyophyllum peltatum* (Dioncophyllaceae)	Root bark	West Ivory Coast	Bringmann *et al.*[60]
	**32**	Mixture of *Triphyophyllum peltatum*,^*a*^*Dioncophyllum thollonii*^*b*^ and *Habropetalum dawei*^c^ (Dioncophyllaceae)	Root and bark^*a*^	West Ivory Coast^*a*^	Bringmann *et al.*[61]
			Twigs^*b, c*^	Gabon^*b*^	
				Sierra Leone^*c*^	
	**33, 34, 35** and **36**	*Triphyophyllum peltatum* (Dioncophyllaceae)	Leaves and twigs	Mt. Nabemba, Congo Republic^*a*^ and	Bringmann *et al.*[62-65]^*a*^
				West Ivory Coast^*b*^	François *et al*. [66]^*b*^
Furoquinolines	**37** and **38**	*Vepris uguenensis* (Rutaceae)	Roots	Baringo District, Kenya	Cheplogoi *et al.*[68]
	**39** and **40**	*Toddalia asiatica* (Rutaceae)	Roots	Ol Ari Nyiro Ranch, Kenya	Gakunju *et al.*[69]
	**41**	*Teclea gerrardii* (Rutaceae)	Root bark	Durban, South Africa	Waffo *et al.*[70]

^a,b^ and ^c^correspond to the respective references.

**Table 2:** Summary of anti-malarial alkaloids derived from the African flora – acridones, amides and cryptolepines

**Compound subclass**	**Isolated metabolites**	**Plant species (Family)**	**Part of plant studied**	**Place of harvest (locality, country)**	**Author, reference**
Acridones	**42**^*a, b, c*^and **43 **^*b*^	*Teclea gerrardii*^*a*^	Root bark^*a*^	Durban, South Africa^*a*^	Waffo *et al.*[70]^*a*^
		*Zanthoxylum leprieurii*^*b*^	Fruits^*b*^	Yaoundé, Cameroon^*b*^	Tchinda *et al.*[71]^*b*^
		*Teclea trichocarpa*^*c*^	Leaves^*c*^		
		(Rutaceae)			
	**44, 45, 46, 47, 48** and **49**	*Teclea trichocarpa* (Rutaceae)	Leaves	Nairobi, Kenya	Wurithi *et al.*[72]
	**50**	*Vepris uguenensis* (Rutaceae)	Roots	Baringo District, Kenya	Cheplogoi *et al.*[68], Kiplimo [73]
Amides	**51**	*Hugonia castaneifolia* (Linaceae)	Root bark	Pugu forest, Tanzania	Baraza *et al.*[74]
	**52**	*Beilschmiedia zenkeri* (Lauraceae)	Bark	Yaoundé, Cameroon	Lenta *et al.*[75]
Cryptolepines	**53**	*Sida acuta* (Malvaceae)	Aerial parts	Ivory Coast	Banzounzi *et al.*[76]
	**53, 54, 55, 56, 57, 58, 59, 60, 61, 62, 63,** and **63′**	*Cryptolepis sanguinolenta* (Periplocaceae)	Stems^*a*^	Mampong-Akwapim, Ghana, Guinea Bissau and other regions	Barku *et al.*[77]^*a*^
			Root bark^*b*^		Cimanga *et al*. [78,79]^*a, b*^
			Roots^*c*^		Ablordeppey *et al*. [80]^*a*^
					Paulo *et al*. [81]^*c*^
					Hadden *et al*. [82]

^a,b^ and ^c^correspond to the respective references.

### Indole alkaloids

Several indole alkaloids, derived from African medicinal plants, have shown interesting *in vitro* anti-malarial activities, among them compounds **1**, **2** and **10** to **19**. Nkunya *et al.* have isolated prenylated indole alkaloids from *Monodora* and *Isolona* species (Annonaceae) growing in Tanzania [47]. According to their report, 6-(3-methyl-but-2-enyl)-1,3-dihydro-indol-2-one (**1**), 3-[6-(3-methyl-but-2-enyl)-1*H*-indolyl]-6-(3- methyl-but-2-enyl)-1*H*-indole or annonidine F (**2**), 1*H*-indole-5-carbaldehyde (**3**), 6-(3-methyl-2-butenyl)-1*H*-indole (**4**), 6-(3-methylbuta-1,3-dienyl)-1*H*-indole (**5**), 6-(4-oxo-but-2-enyl)-1*H*-indole (**6**) and 3-geranylindole (**7**) were isolated from *Monodora angolensis* while 3-(1,1-dimethyl-but-2-enyl)-5-(3-methyl-but-2-enyl)-1*H*-indole or caulidine A (**8**), 4-[3-(1,1-dimethyl-but-2-enyl)-1*H*-indol-5-yl]-but-3-en-2-one or caulidine B (**9**), 5-(3-methyl-2-butenyl)-1*H*-indole and 5-(3-methylbuta-1,3-dienyl)-1*H*-indole were obtained from *Isolona cauliflora*. The compounds with the most promising, measured, anti-malarial activities were **1** and **2**, both having *in vitro* anti-malarial activities against the multidrug resistant strain K1 of *P. falciparum* (IC_50_ = 21 μg mL^-1^ for each compound). Moreover, their measured cytotoxicities, in the brine shrimp test were IC_50_ = 4.08 and 5.28 μg mL^-1^, respectively.

The compound 17-*O*-acetyl,10-hydroxycorynantheol (**10**) was isolated from *Strychnos usambarensis* (harvested in Rwanda), along with isostrychnopentamine (**18**), the main alkaloid responsible for the anti-plasmodial activity of the plant, by Cao *et al*. [48]. The study showed that compound **10** is one of the most promising, monomeric indole alkaloids known to date, showing an *in vitro* activity against *P. falciparum* close to 5 μM and a high selectivity.

Indoles with interesting anti-malarial properties have also been derived from two plant species growing in Cameroon: *Penianthus longifolius* and *Glossocalyx brevipes*[49,50]. Bilda *et al.* have isolated palmitine (**11**) and jatrorrhizine (**12**) from the stem bark of *Penianthus longifolius*. Compounds **11** and **12** showed promising *in vitro* activities on various strains of *P. falciparum* with IC_50_ values ranging from 0.28 to 0.35 μg mL^-1^[49], meanwhile Mbah *et al.* isolated liriodenine (**13**) from *Glossocalyx brevipes* (Siparunaceae), which exhibited anti-malarial activity against the D-6 drug sensitive strain from Sierra Leone and the NF54 strain with IC_50_ values of 2.37 μM and 1.32 μM, respectively [50].

From the plant species growing in Nigeria, fagaronine (**14**) and alstonine (**15**) were derived from *Fagara zanthoxyloides* (Rutaceae) and *Picralima nitida* (Apocynaceae) respectively [51,52]. While fagaronine (**14**) inhibited *P. falciparum* growth *in vitro* at IC_50_ = 0.018 μg mL^-1^, alstonine (**15**) has been noted to be the most active indole alkaloid derived from *Picralima nitida*[53]. It is noteworthy that indole and dihydroindole alkaloids are common in *Picralima nitida* growing in Nigeria, the major constituents including akuammiline, akuammidine, akuammine, akuammigine, akuammicine, picraline, and alstonine [54]. Some of the aforementioned alkaloids have exhibited *in vitro* anti-malarial activity against *P. falciparum* comparable to chloroquine and quinine [52], the IC_50_ values varying from 0.01 to 0.9 μg mL^-1^[53].

Frédérich *et al.* examined the roots and leaves of *Strychnos usambarensis* (Loganiaceae) growing in Rwanda [55,56]. Four potent anti-malarial bisindole alkaloids; 10′-hydroxyusambarensine (**16**), strychnopentamine (**17**), isostrychnopentamine (**18**) and chrysopentamine (**19**) have been isolated. Compounds **17** to **19** showed interesting anti-malarial activities against chloroquine-sensitive line (FCA 20) from Ghana (IC_50_ values from 117 to 579 nM), against moderately chloroquine-resistant line (FCB1-R) from Colombia (IC_50_ values from 107 to 550 nM) and against chloroquine-resistant line (W2) from Indochina (Laos) (IC_50_ values from 145 to 507 nM). The results of anti-plasmodial activities were comparable to those of the anti-malarial drugs quinine and chloroquine, which is indicative of the absence of cross-resistance with chloroquine. Meanwhile, compound **16** had moderate *in vitro* activity against two strains of *P. falciparum*.

### Naphthoisoquinolines

These compounds are characterized by the C5/C8’ linkage between the naphthalene and the isoquinoline portions of these alkaloids (Figure 3). They have been isolated from *Ancistrocladus* (Acistrocladaceae), *Triphyophyllum*, *Dioncophyllum,* and *Habropetalum* (Dioncophyllaceae) species. The chemical significance of naphthylisoquinoline alkaloids rests on their unique structure and their biological activities [45,46].

The anti-malarial properties of some of these species have been investigated by Bringmann *et al.*[57-66]. Regarding the Acistrocladaceae-derived naphthoisoquinolines, compounds **20** to **24**, derived from the stems and leaves of *Ancistrocladus robertsoniorum* growing in Kenya, exhibited moderate anti-malarial activities (IC_50_ values from 2.0 to 15.9 μM) against the K-1 and NF54 strains of *P. falciparum*[57], meanwhile the Tanzanian species, *Ancistrocladus tanzaniensis*, gave compounds **25** to **29** with IC_50_ values ranging from 0.1 to 3.6 μg mL^-1^ against the K1 strain and between 1.9 and 34.1 μg mL^-1^ against the 3D7 strain [58]. Habropetaline A (**30**) and 5′-*O*-demethyl-dioncohylline A (**31**) were derived from the roots of *Triphyophyllum peltatum*, harvested in the Parc de Taï, in west Ivory Coast [59,60]. Both naphthoisoquinolines exhibited interesting anti-plasmodial activities against drug-sensitive and drug-resistant strains of the parasite. Habropetaline A (**30**) showed very good effect against *P. falciparum*, without cytotoxicity, with respective IC_50_ values of 5.0 and 2.3 ng mL^-1^ for the strains K1 (chloroquine and pyrimethamine resistant) and NF54 (sensitive to all known drugs). Compound **30** was almost as active as artemisinin (K1: 1.2 ng mL^-1^, NF54: 1.2 ng mL^-1^) and is known to be one of the most potent NPs used against *P. falciparum*[59]. On the other hand, 5′-*O*-demethyl-dioncophylline A (**31**) showed improved *in vitro* anti-malarial activity (IC_50_ = 0.340 μg mL^-1^) against the erythrocytic forms of *P. falciparum*[60]. Jozipeltine A (**32**), the dimer of the highly hydroxylated naphthylisoquinoline alkaloid dioncopeltine A (**36**), was derived from a mixture of root and bark of *Triphyophyllum peltatum* and *Dioncophyllum thollonii*, along with twigs of *Habropetalum dawei* (Dioncophyllaceae), harvested from different regions on the continent [61]. Although this compound showed some *in vitro* anti-plasmodial activity against *P. falciparum* (K1 = 875 ng mL^-1^, NF54 = 2530 ng mL^-1^), it is significantly less active than its monomeric precursor, dioncopeltine A (**36**) (K1 = 4.8 ng mL^-1^, NF54 = 3.3 ng mL^-1^). This observation could lead to the conclusion that only naphthoisoquinolines containing one phenolic OH group each (such as dioncophylline A (**36**) and ancistrocladine (**28**)), could easily undergo the required dimerization reaction, implying that doubling of the number of free OH groups would increase the anti-plasmodial activity [61]. Dioncophyllines A (**33**), B (**34**) and C (**35**) and dioncopeltine A (**36**) were also active in the *in vivo* rodent model [66], with dioncophylline C (**35**) exhibiting a 50% effective dosage (ED_50_) of 10.71 mg kg^-1^ day^-1^. Four daily treatments with 50 mg kg^-1^ day^-1^ were needed to achieve radical cure, one oral dose being sufficient to kill 99.6% of the parasites. Intravenous application of dioncophylline C was shown to be even more effective, with an ED_50_ of 1.90 mg kg^-1^ day^-1^ and no noticeable toxic effects. Compound **35** also suppressed more established *Plasmodium berghei* infections when orally applied at day 3 after infection. It should be mentioned that rodent malaria is a well-known animal model for testing new compounds and plant extracts. However, trial in human being is decisive to identify a “hit” as “a real hit”; and this is a good way to assess toxicity and safety. Both dioncopeltine A (**36**) and dioncophylline C (**35**) were active against the chloroquine-resistant *P. berghei* Anka CRS parasites. The naphthoisoquinolines are also known to exhibit other biological activities, e.g. dioncophylline A (**33**), is the main cytotoxin in *Ancistrocladus letestui*[67]. The above observations all point to the fact that naphthylisoquinoline alkaloids are promising lead compounds for the development of anti-malarial drugs.

### Furoquinolines

This subclass of alkaloids is easily identified with the *Vepris*, *Toddalia* and *Teclea* genera of the Rutaceae family. From the roots of *Vepris uguenensis*, Cheplogoi *et al*. isolated flindersiamine (**37**) and maculosidine (**38**) [68]. Although compound **37** lacked anti-malarial efficacy against all tested strains, maculosidine (**38**) exhibited moderate anti-malarial activity against two strains of *P. falciparum*, with IC_50_ values of 29.2 and 40.4 μg mL^-1^ against the chloroquine-susceptible 3D7 and the chloroquine-resistant FCM29 strains respectively. Nitidine (**39**) has been derived from the roots of *Toddalia asiatica* harvested in Kenya and modified to yield the reduced derivative 5,6-dihydronitidine (**40**) [69]. Even though nitidine is mostly known for its potential anticancer properties, the investigations of Gakunju *et al*. showed the alkaloidal extract of the roots of this plant to have high activity against the chloroquine-resistant K39 strain of *P. falciparum*, with an IC_50_ value of 0.04 μg mL^-1^. Further phytochemical analysis on the extract by these authors yielded nitidine as a major compound. *In vitro* screening against the K39 strain of *P. falciparum* revealed that nitidine exhibited high anti-plasmodial activity, with an IC_50_ of 0.045 μg mL^-1^, in addition to its known cytotoxic property. In order to remove toxicity, synthetic modification led to 5,6-dihydronitidine (**40**), with a much weaker anti-malarial activity (IC_50_ of 1.03 μg mL^-1^, 23 times weaker than nitidine). Evoxine (**41**), derived from *Teclea gerrardii* (Rutaceae) harvested from Durban, South Africa, displayed moderate anti-plasmodial activity against the CQS D10 strain of *P. falciparum*, with IC_50_ value 24.5 μM [70].

### Acridones

The most promising anti-plasmodial acridones derived from the African flora include arborinine (**42**), xanthoxoline (**43**), normelicopicine (**44**), skimmianine (**45**), dictamnine (**46**), melicopicine (**47**), tecleanthine (**48**) and 6-methoxytecleanthine (**49**), shown in Figure 5, isolated from *Teclea* and *Zanthoxylum* species. Compound **42** was derived from *Teclea gerrardii*, *Zanthoxylum leprieurii* and *Teclea trichocarpa* (Rutaceae) and has shown anti-plasmodial activity against 3D7 strains (IC_50_ = 4.5 μg mL^-1^), almost equally active as compound **43** (IC_50_ = 4.6 μg mL^-1^) [70,71]. Compounds **44** to **49** showed moderate activity against the chloroquine-sensitive HB3 and the chloroquine-resistant K-1 strains of *P. falciparum*, with respective anti-plasmodial IC_50_ values of 14.7, 9.3, 59.0, 53.0, and 56.9 μM [72]. Compound **50** (maculosidine), derived from *Vepris uguenensis* (Rutaceae), exhibited anti-malarial activities at 13.8 and 40.4 μg mL^-1^ against the 3D7 (chloroquine susceptible, CQS) and FCM29 (chloroquine resistant, CQR) strains of *P. falciparum*, respectively [73].

### Amides

*N*-isobutyldeca-2,4-dienamide (**51**) and pipyahyine (**52**), Figure 6, are two amides respectively derived from *Hugonia castaneifolia* (Linaceae) and *Beilschmiedia zenkeri* (Lauraceae) [74,75]. It has been shown that compound **51** had moderate anti-plasmodial activity against the K-1 strain of *P. falciparum*, with an IC_50_ value of 5.4 μg mL^-1^[74], while compound **52** showed activity against the chloroquine-resistant W2 strain of *P. falciparum*, with an IC_50_ value of 3.7 μM [75].

### Cryptolepines

Cryptolepine (**53**), derived from *Sida acuta* (Malvaceae), growing in Ivory Coast, has shown very good anti-malarial activity [76]. According to Banzouzi *et al*., the IC_50_ values obtained for the extracts from this plant ranged from 3.9 to 5.4 μg mL^-1^. Purification of this active fraction led to the identification of cryptolepine (**53**) as the active anti-plasmodial constituent of the plant. Compound **53** exhibited IC_50_ values against the chloroquine-sensitive strain (respectively 0.13 and 0.17 μg mL^-1^ after 24 and 72 hours) from Nigeria and the Fcm29 chloroquine-resistant strain (respectively 0.17 and 0.17 μg mL^-1^ after 24 and 72 hours) from Cameroon. Cryptolepine derivatives (**54** to **63**), Figure 7, isolated from the stems, roots and root bark of *Cryptolepis sanguinolenta* (Periplocaceae) growing in diverse regions in Africa, have also exhibited potent anti-malarial properties [77-80].

Cimanga *et al*. assessed three different extracts and four alkaloids from the root bark of *Cryptolepis sanguinolenta in vitro* against *P. falciparum* D-6 (chloroquine-sensitive strain), K-1, and W-2 (chloroquine-resistant strains). Cryptolepine (**53**) and its hydrochloride salt, 11-hydroxycryptolepine (**56**), and neocryptolepine (**61**) showed strong anti-plasmodial activity against *P. falciparum* chloroquine-resistant strains. Quindoline (**54**) was less active. The highest activity was obtained with cryptolepine (**53**). *In vivo* tests on infected mice showed that cryptolepine exhibited a significant chemosuppressive effect against *Plasmodium yoelii* and *Plasmodium berghei*, while cryptolepine (**53**) had the same effect against *P. yoelii* only. Compounds **54** and **56** did not show activity in this *in vivo* test system [79].

Another study by Paulo *et al*. on the roots of *Cryptolepis sanguinolenta* harvested from Guinea-Bissau led to the isolation of cryptolepinoic acid (**62**) and methyl cryptolepinoate (**63**) in addition to **53**, **54** and **56** from the ethanol and chlorophorm extracts of the leaves [81]. The isolated compounds and extracts were tested *in vitro* against *P. falciparum* K1 (multidrug-resistant strain) and T996 (chloroquine-sensitive clone). All extracts had 90% inhibition of *P. falciparum* K1 growth at concentrations <23 μg mL^-1^. Cryptolepine (**53**) was the most active alkaloid tested with IC_50_ values (0.23 μM to K1; 0.059 μM to T996), compared to chloroquine (0.26 μM to K1; 0.019 μM to T996). The indolobenzazepine alkaloid cryptoheptine (**57**) was the second most active with IC_50_ values of 0.8 μM (K1) and 1.2 μM (T996). Cryptolepinoic acid (**62**) showed no significant activity while its ethyl ester derivative (**63′**) was active against *P. falciparum* K1 (IC_50_ = 3.7 μM). All the indoloquinoline alkaloids showed cross-resistance with chloroquine but not the indolobenzazepine cryptoheptine (**57**). It was noticed that alkaloids with weakly basic characteristics were active whereas other structurally related alkaloids with different acid–base profiles were inactive. These observations are in agreement with the anti-malarial mechanism of action for quinolines. According to Hadden *et al*., the unusual incorporation of the isopropyl group at the 11-position of the indolo [3,2-*b*] quinoline nucleus in 11-isopropylcryptolepine (**56**) is suggestive of a mixed biosynthetic origin for the alkaloid [82].

### Terpenoids

Terpenoids with most promising anti-malarial properties are summarized in Tables 3, 4 and 5 (according to their subclasses), while the chemical structures are shown in Figures 8, 9, 10, 11, 12, 13, 14, 15, 16, 17, 18 and 19.

**Table 3:** Summary of anti-malarial terpenoids derived from the African flora, part 1: clerodanes, labdanes, limonoids, bisnorterpenes and acyclic triterpenes

**Compound subclass**	**Isolated metabolites**	**Plant species (Family)**	**Part of plant studied**	**Place of harvest (locality, country)**	**Author, reference**
Clerodane and labdane diterpenoids	**64** to **70**	*Nuxia sphaerocephala* (Loganiaceae)	Leaves	Ankazobe, Madagascar	Mambu *et al.*[83]
	**71** to **74**	*Aframomum latifolium or sceptrum* (Zingiberaceae)	Fruits and Leaves	Acrra, Ghana	Duker-Eshun *et al.*[84]
	**75, 76** and **77**	*Turreanthus africanus* (Meliaceae)	Seeds	Mt. Cameroon, Cameroon	Ngemenya *et al.*[85]
	**78, 79, 80,** and **81**	*Aframomum zambesiacum* (Zingiberaceae)	Seeds	Nyasoso, Cameroon	Kenmogne *et al*. [86]
	**74**^*a, b*^**, 82**^*a*^ and **83**^*b*^	*Aframomum*	Seeds^*a*^	Mogbi, Cameroon^*a*^	Ayimele *et al*. [87]^*a*^
		*escapum*^*a*^	Fruits and leaves^*b*^		
		(Zingiberaceae) *Aframomum latifolium* and *sceptrum*^*b*^		Acrra, Ghana^*b*^	Duker-Eshun *et al.*[84]^*b*^
		(Zingiberaceae)			
	**84, 85, 86,** and **87**	*Aframomum arundinaceum* (Zingiberaceae)	Seeds	Maha, Cameroon	Wabo *et al.*[88]
Limonoids	**88**	*Vepris uguenensis* (Rutaceae)	Roots	Baringo District, Kenya	Cheplogoi *et al.*[73], Kiplimo [73]
	**89, 90, 91, 92,** and **93**	*Khaya grandifoliola* (Meliaceae)	Bark and seeds	Foumban, Cameroon	Bickii *et al.*[89]
	**94** and **95**	*Entandrophragma angolense* (Meliaceae)	Stem bark	Awae forest reserve, Cameroon	Bickii *et al.*[90]
	**96, 97, 98,** and **99**	*Ekebergia capensis* (Zingiberaceae)	Stem bark	Mt Kenya, Kenya	Murata *et al.*[91]
Bisnorterpenes	**100, 101, 102,** and **103**	*Salacia madagascariensis* (Celastraceae)	Roots	Tanzania	Thiem *et al.*[92]
Acyclic triterpenes	**104, 105, 106,** and **107**	*Ekebergia capensis* (Zingiberaceae)	Stem bark	Mt Kenya, Kenya	Murata *et al.*[91]
	**108**	*Aframomum escapum* (Zingiberaceae)	Seeds	Mogbi, Cameroon	Ayimele *et al*. [87]
					Lopez et al. [93]

^a,b^ and ^c^correspond to the respective references.

**Table 4:** Summary of anti-malarial terpenoids derived from the African flora, part 2: cassane furanoditerpenes, abietane diterpenes and sesquiterpenes

**Compound subclass**	**Isolated metabolites**	**Plant species (Family)**	**Part of plant studied**	**Place of harvest (locality, country)**	**Author, reference**
Cassane furanoditerpenes	**109** and **110**	*Caesalpinia volkensii* (Leguminosae)	Root bark	Gatamaiyo forest, Kenya	Ochieng *et al.*[94]
Abietane diterpenes	**111** and **112**	*Plectranthus hadiensis* (Lamiaceae)	Leaves	South Africa	van Zyla *et al.*[95]
	**113**	*Plectranthus lucidus* (Lamiaceae)	Leaves	South Africa	van Zyla *et al.*[95]
	**114**	*Plectranthus ecklonii* (Lamiaceae)	Leaves	South Africa	van Zyla *et al.*[95]
	**115, 116** and **117**	*Plectranthus purpuratus* (Lamiaceae)	Leaves	South Africa	van Zyla *et al.*[95]
	**118**	*Fuerstia africana* (Lamiaceae)	Aerial parts	Ngong Hills, Kenya	Koch *et al*. [96]
	**119**	*Hoslundia opposita* (Lamiaceae)	Root bark	Tanzania	Achenbach *et al.*[97]
	**120**	*Hyptis suaveolens* (Lamiaceae)	Leaves	Southeastern Nigeria	Chukwujekwu *et al.*[98]
Sesquiterpenes and sesquiterpene lactones	**121, 122, 123,** and **124**	*Vernonia amygdalina* (Asteraceae)	Leaves	Mahale National Mountains Park, Tanzania	Ohigashi *et al.*[99]
	**125**	*Vernonia brachycalyx* (Asteraceae)	Leaves	Machakos District, Kenya	Oketch-Rabah *et al*. [100]
	**126**	*Ajuga remota* (Lamiaceae)	Aerial parts	Nairobi, Kenya	Kuria *et al*. [101]
	**127, 128** and **129**	*Reneilmia cincinnata* (Zingiberaceae)	Fruits	Bafut, Cameroon	Tchuendem *et al*. [102]
	**130** and **131**	*Acanthospermum hispidum* (Asteraceae)	Flowers, leaves and stems	Danto/Porto-Novo, Benin	Ganfon *et al*. [103]
	**132, 133, 134,** and **135**	*Vernonia angulifolia* (Asteraceae)	Aerial parts	University of KwaZulu-Natal, South Africa	Pedersen *et al*. [104]
	**136**	*Dicoma tomentosa* (Asteraceae)	Whole plant	Poun, Burkina Faso	Jansen *et al*. [105]
	**137**	*Artemisia annua* (Asteraceae)	Seeds	Kjenzi (Bugarama), Burundi	Reale *et al*. [106]
	**138**	*Dicoma anomala subsp. gerrardii* (Asteraceae)	Root stocks	Brits region, South Africa	Becker *et al*. [107]
	**139**	*Tithonia diversifolia* (Asteraceae)	Aerial parts	São Tomé and Príncipe islands	Goffin *et al.*[108]
	**140**	*Scleria striatinux* (Cyperaceae)	Roots	Oku, Cameroon	Efange *et al.*[109]
Coloratane sesquiterpenes	**141** to **148**	*Warburgia ugandensis* (Canellaceae)	Stem bark	Dello Menna, Ethiopia	Wube *et al.*[110]

**Table 5:** Summary of anti-malarial triterpenoids derived from the African flora, part 3: Beilshmiedic acid derivatives and pentacyclic triterpenes

**Compound subclass**	**Isolated metabolites**	**Plant species (Family)**	**Part of plant studied**	**Place of harvest (locality, country)**	**Author, reference**
Beilshmiedic acid derivatives	**149, 150, 151, 152,** and **153**	*Beilschmiedia cryptocaryoides* (Lauraceae)	Bark	Ranomafana-Ifanadiana, Madagascar	Talontsi *et al.*[111]
Pentacyclic triterpenes	**154**	*Schefflera umbellifera* (Araliaceae)	Leaves	South Africa	Mthembu [112]
	**155**	*Maytenus senegalensis* (Celastraceae)	Root bark	Eastern region of Sudan	Khalid *et al*. [113]
	**156, 157, 158, 159,** and **160**	*Nuxia sphaerocephala* (Loganiaceae)	Leaves	Ankazobe, Madagascar	Mambu *et al.*[83]
	**161** and **162**	*Hymenocardia acida* (Phyllanthaceae)	Bark	Chad	Mahmout *et al.*[114]
	**161**	*Cassia siamea* (Fabaceae)	Stems	Otu (Oyo State), Nigeria	Ajaiyeoba *et al.*[115]
	**163** and **164**	*Entandrophragma angolense* (Meliaceae)	Stem bark	Awae forest reserve, Cameroon	Bickii *et al.*[90]
	**165**	*Hypericum lanceolatum* (Hypericaceae)	Stem bark	Mt. Bamboutos, Cameroon	Zofou *et al*. [116]
	**166**	*Psorospermum glaberrimum* (Hypericaceae)	Stem bark	Ekombitié, Cameroon	Lenta *et al*. [117]
	**167**	*Baillonella toxisperma* (Sapotaceae)	Stem bark	Korup forest reserve, Cameroon	Mbah *et al*. [118]
	**168**	*Kigelia africana* (Bignoniaceae)	Stem bark	Bandjoun, Cameroon	Zofou *et al*. [119]
	**169, 170** and **171**	*Cogniauxia podolaena* (Cucurbitaceae)	Stem bark	Congo	Banzouzi *et al*. [120]

**Figure 8:**
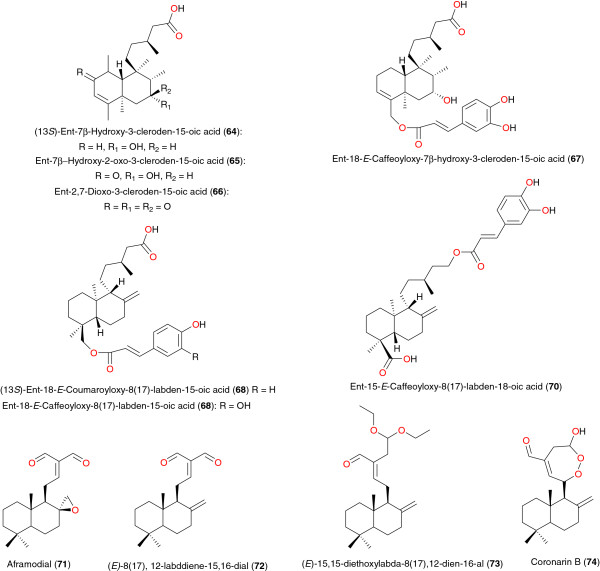
Clerodane and labdane diterpenoids from African medicinal plants with anti-malarial activity - I.

**Figure 9:**
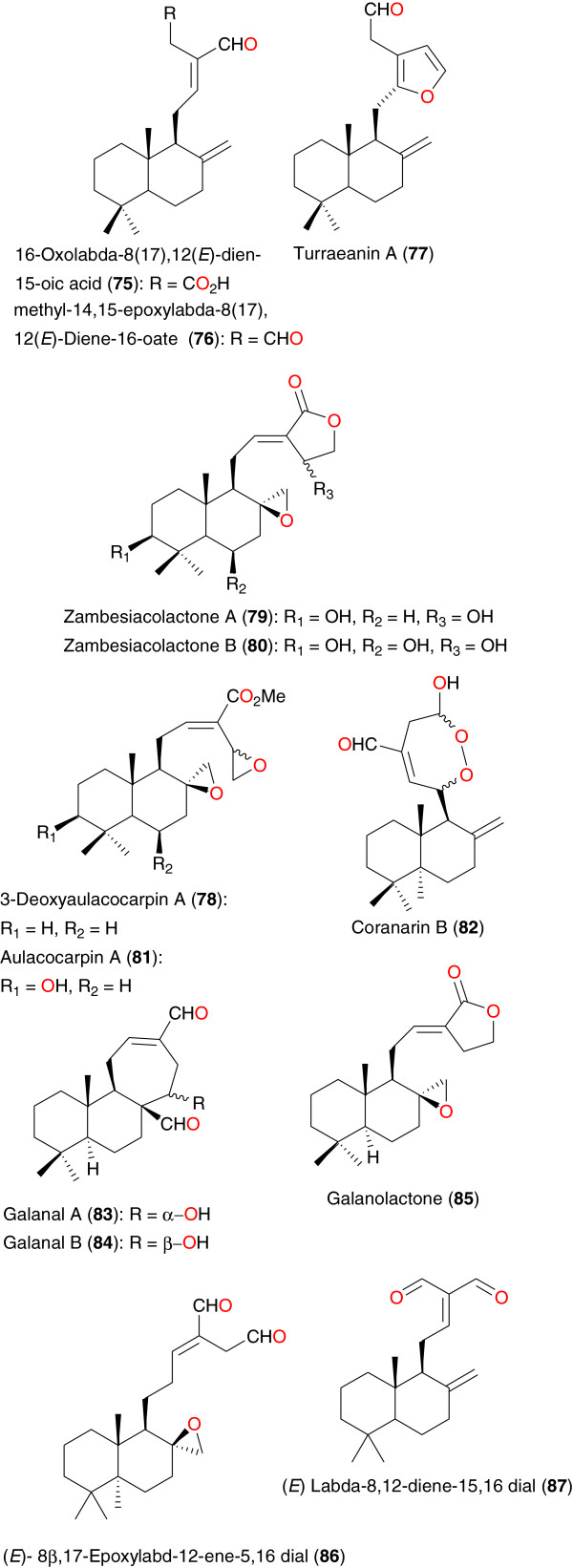
Clerodane and labdane diterpenoids from African medicinal plants with anti-malarial activity – II.

**Figure 10:**
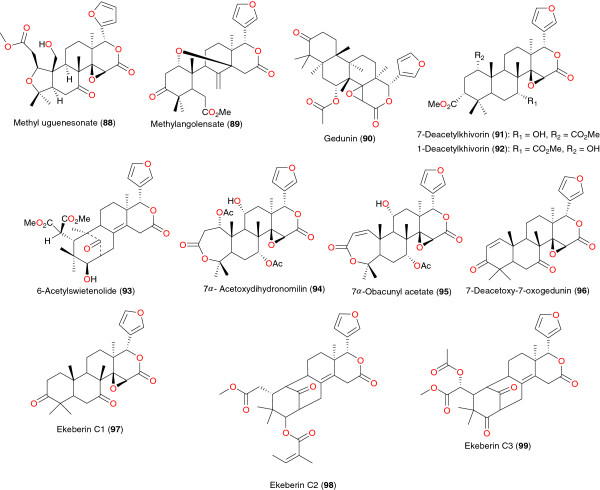
Limonoids from African medicinal plants with antiplasmodial activity.

**Figure 11:**
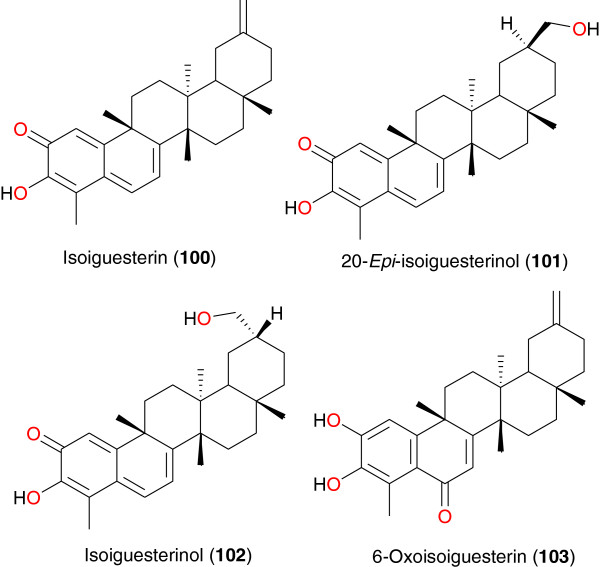
Bisnorterpenes from African medicinal plants with anti-malarial activity.

**Figure 12:**
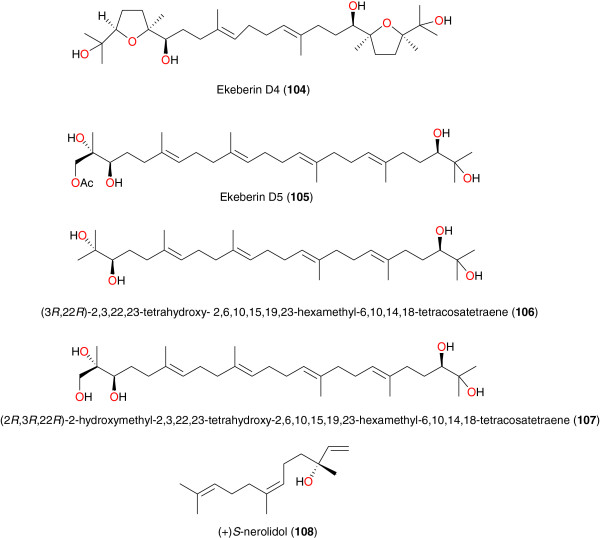
Anti-malarial acyclic triterpenes from African medicinal plants.

**Figure 13:**
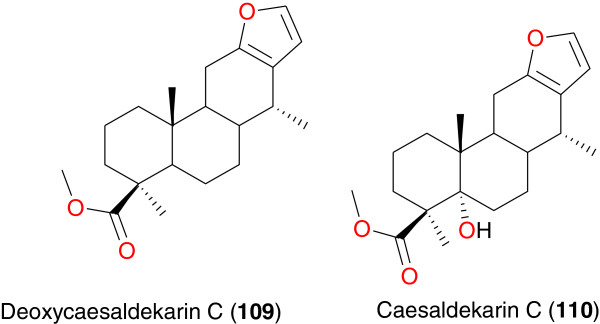
Promising anti-malarial cassane furanoditerpenes from African medicinal plants.

**Figure 14:**
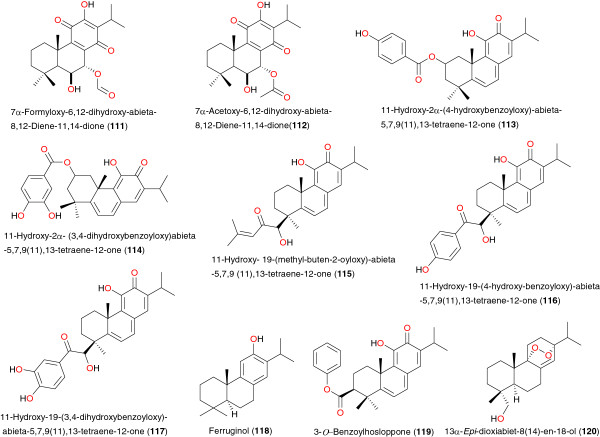
Promising anti-malarial abietane diterpenes from African medicinal plants.

**Figure 15:**
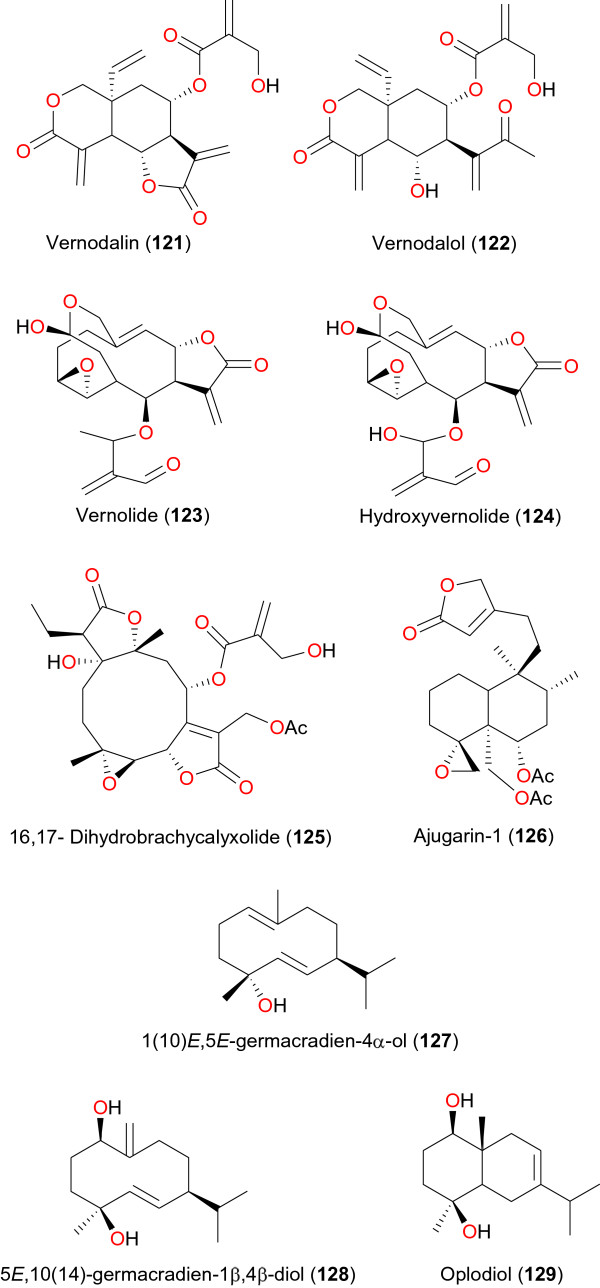
Some anti-malarial sesquiterpenes and sesquiterpene lactones from African medicinal plants - I.

**Figure 16:**
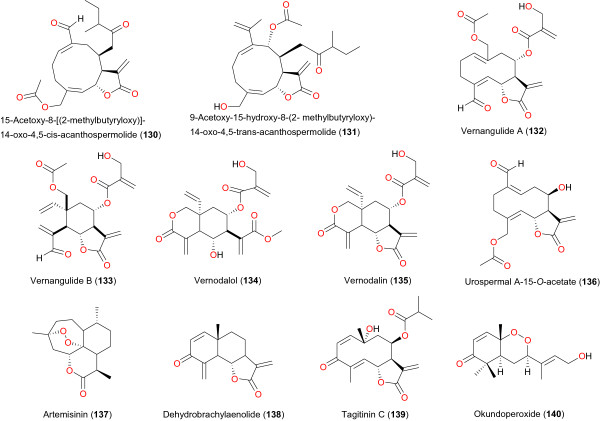
Some anti-malarial sesquiterpenes and sesquiterpene lactones from African medicinal plants - II.

**Figure 17:**
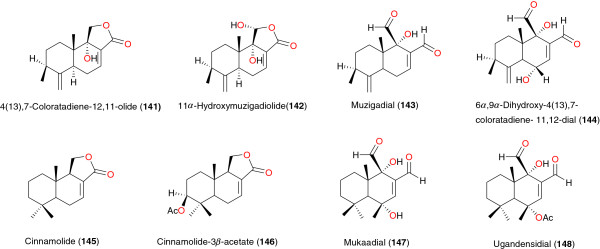
Coloratane sesquiterpenes from African medicinal plants with anti-malarial activity.

**Figure 18:**
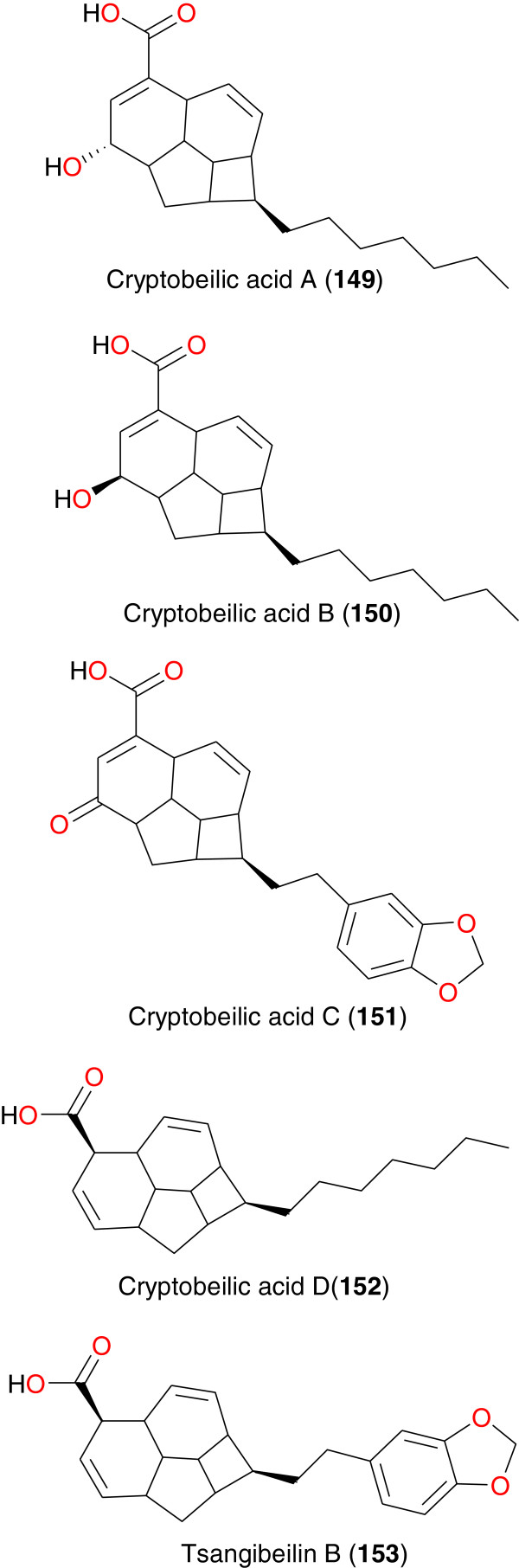
Beilshmiedic acid derivatives from African medicinal plants with anti-malarial activity.

**Figure 19:**
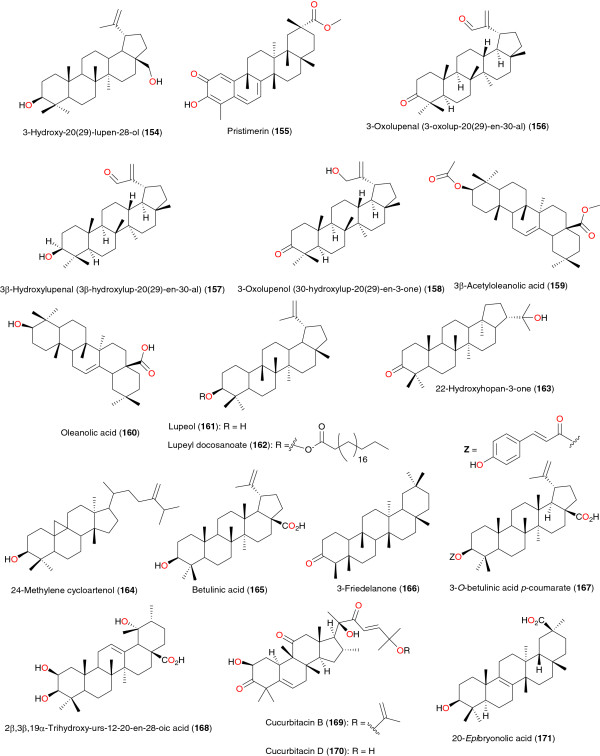
Pentacyclic triterpenes from African medicinal plants with antiplasmodial activity.

### Clerodane and labdane diterpenoids

Mambu *et al*. isolated the clerodanes (13*S*)-ent-7β-hydroxy-3-cleroden-15-oic acid (**64**), ent-7β-hydroxy- 2-oxo-3-cleroden-15-oic acid (**65**), ent-2,7-dioxo-3-clero-den-15-oic acid (**66**) and ent-18-(*E*)-caffeoyloxy-7β-hydroxy-3-cleroden-15- oic acid (**67**) and the labdanes (13*S*)-ent-18-(*E*)-coumaroyloxy-8(17)-labden-15-oic acid (**68**), ent-18-(*E*)-caffeoyloxy-8(17)-labden-15-oic acid (**69**) and ent-15-*E*-caffeoyloxy-8(17)-labden-18-oic acid (**70**) from *Nuxia sphaerocephala*, growing in Ankazobe, 100 km from Antananarivo, Madagascar [83]. The compounds showed *in vitro* anti-plasmodial activities against the FcB1 *P. falciparum* strain, with respective IC_50_ values 14.6, 4.3, 8.0, 7.3, 11.4, 21.0, 16.0 μg mL^-1^. Duker-Eshun *et al*. obtained aframodial (**71**), (*E*)-8(17), 12-labddiene-15,16-dial (**72**), (*E*)-15,15-diethoxylabda-8(17),12-dien-16-al (**73**) and coronarin B (**74**) from the fruits and leaves of *Aframomum latifolium* or *Aframomum sceptrum* (Zingiberaceae) harvested in Accra, Ghana. Respective IC_50_ values of 25, 48, 24, and 26 μM against the chloroquine-sensistive strain (3D7) were obtained for these compounds [84]. Anti-plasmodial activites were also obtained for labdanes 16-oxolabda-8(17),12(*E*)-dien- 15-oic acid (**75**), methyl-14,15-epoxylabda-8(17), 12(*E*)-diene-16-oate (**76**) and turraeanin A (**77**), from the seeds of *Turreanthus africanus* (Meliaceae), a plant generally used in preparations against fevers and malaria in ATM [85]. Compound **75** showed the highest anti-plasmodial activity (IC_50_ of 26 μg mL^-1^) on chloroquine-sensitive *P. falciparum* F 32, *in vitro*, while compounds **76** and **77** rather had moderate activities [85]. Other anti-malarial labdanes of *Aframomum* sp. include 3-deoxyaulacocarpin A (**78**), zambesiacolactones A (**79**) and B (**80**) and aulacocarpin A (**81**) from seeds of *Aframomum zambesiacum*[86]; coranarin B (**82**) from the seeds of *Aframomum escapum*[87]; galanal A (**83**) from the leaves of *Aframomum sceptrum*[84]; galanal B (**84**), galanolactone (**85**), (*E*)- 8β,17-epoxylabd-12-ene-5,16 dial (**86**) and (*E*) labda-8,12-diene-15,16 dial (**87**) from the seeds of *Aframomum arundinaceum*[88]. Among these compounds, compound **76** (3-deoxyaulacocarpin A), derived from *Aframomum zambesiacum*, was both the least polar and the most active compound, with an IC_50_ of 4.97 μM (1.73 μg/ml) [86].

### Limonoids

Limonoids with good anti-plasmodial activities have been isolated from *Vepris uguenensis* (Rutaceae), harvested in Kenya [68,73], as well as from *Khaya grandifoliola* (Meliaceae) and *Entandrophragma angolense* (Meliaceae) harvested in Cameroon [89,90]. The chemical structures are shown in Figure 10. Methyl uguenesonate (**88**) from *Vepris uguenensis* displayed mild activity, with IC_50_ values of 10.4 and 13.8 μg mL^-1^, against the CQS and CQR strains of *P. falciparum*, respectively [68,73]. The bark and seed extracts of *Khaya grandifoliola* (Meliaceae), a plant species widely used in the Central African subregion to treat various ailments, including malaria have also been investigated. Seven limonoids were isolated, among which five were significantly active (with IC_50_ values ranging between 1.25 and 9.63 μg mL^-1^). These include: methylangolensate (**89**); gedunin (1.25 μg mL^-1^) (**90**); 7-deacetylkhivorin (**91**); 1-deacetylkhivorin (**92**) and 6-acetylswietenolide (**93**). The same authors also investigated the stem bark of *Entandrophragma angolense* (Meliaceae) and isolated known limonoids with anti-malarial activity; 7α-acetoxydihydronomilin (**94**), 7α-obacunylacetate (**95**) and methylangolensate (**89**). Compounds **89** and **95** were considerably active against *P. falciparum* W2 with respective IC_50_ values of 2.0 and 19.5 μg mL^-1^.

Among the four limonoids derived from the stem bark of *Ekebergia capensis*, namely 7-deacetoxy-7-oxogedunin (**96**), ekeberins C1 (**97**), C2 (**98**) and C3 (**99**), only compound **96** exhibited significant activity against the chloroquine-susceptible FCR-3 strain of *P. falciparum*, with an IC_50_ of 6 μM, but it lacked efficacy against the chloroquine-resistant K-1 strain [91].

### Bisnorterpenes

Bisnorterpenes with interesting anti-plasmodial properties were purified from the roots of *Salacia madagascariensis* (Celastraceae), a shrub found in East Africa whose roots are used in the treatment of malaria, fever and menorrhagia specifically in Tanzania [92]. This plant is a rich source of bisnortriterpenes with potent antiprotozoal activity [45]. Four bisnortriterpenes; isoiguesterin (**100**), 20-*epi*-isoiguesterinol (**101**), isoiguesterinol (**102**) and 6-oxoisoiguesterin (**103**), were reported from the roots of this plant [92], Figure 11. However, only the first two showed high activity, with respective IC_50_ values of 200 and 68 ng mL^-1^ against the D6 strain of *P. falciparum*, and 170 and 68 ng mL^-1^ (against the W2 strain of *P. falciparum*), respectively.

### Acyclic triterpenes

The most active acyclic triterpenes have been found in the stem back of *Ekebergia capensis* (Zingiberaceae) by Murata *et al*. [91]. Four triterpenes from the stem bark of this species, comprising two new acyclic triterpenoids, namely ekeberin D4 (**104**) and D5 (**105**) and two known ones (3*R*,22*R*)-2,3,22,23-tetrahydroxy-2,6,10,15,19,23-hexamethyl-6,10,14,18-tetracosatetraene (**106**) and (2*R*,3*R*,22*R*)-2-hydroxymethyl-2,3,22,23-tetrahydroxy- 2,6,10,15,19,23-hexamethyl-6,10,14,18-tetracosatetraene (**107**) have been identified, Figure 12. Compounds **106** and **107** exhibited moderate anti-malarial activity against the FCR-3 strain of *P. falciparum*, with IC_50_ values of 55 and 18 μM respectively, in addition to the good activities against the chloroquine-resistant K-1 strain, against which they had respective IC_50_ values of 7 and 59 μM. The triterpene **104** lacked efficacy, while **105** had an IC_50_ of 137 μM against the same parasite [91]. (+) *S*-nerolidol (**108**) isolated from the seeds of *Aframomum escapum*[87], is an important constituent of essential oils used in the treatment of malaria. This compound is also found in *Artemisia herba alba* and in lemon grass, and is able to arrest development of the intraerythrocytic stages of the parasite. Compound **108** was identified as the active constituent leading to 100% growth inhibition at the schizont stage [93].

### Cassane furanoditerpenes

Ochieng *et al*. isolated the cassane furanoditerpenes; deoxycaesaldekarin C (**109**) and caesaldekarin C (**110**) from the the chloroform and ethyl acetate extracts of the root bark of *Caesalpinia volkensii* from Kenya (Figure 13). These two compounds have exhibited antinociceptive and anti-plasmodial activities [94]. The anti-plasmodial activities were evaluated against chloroquine-sensitive (D6) and chloroquine-resistant (W2), with respective IC_50_ values of 25.67 and 30.33 μg mL^-1^ for compound **109** and respective IC_50_ values of 34.44 and 30.69 μg mL^-1^ for compound **110**. The results however demonstrated that *Caesalpinia volkensii* and other members of this genus contain cassane furanoditerpenes, which play a role in the medicinal properties of their plant root barks. The antinociceptive action in chemical models of nociception in mice suggests that the root bark extract and the active principles (furanoditerpenes) represent potential therapeutic options for the management of pain related ailments and not malaria [94].

### Abietane diterpenes

The anti-malarial properties of *Plectranthus* sp. (Lamiaceae), harvested in South Africa, have been determined by van Zyla *et al*. [95]. Seven abietane diterpenes (see Figure 14) were isolated from *Plectranthus hadiensis*, *Plectranthus lucidus*, *Plectranthus ecklonii*, *Plectranthus purpuratus* subsp. *purpuratus* and *Plectranthus purpuratus* subsp. *tongaensis*; 7α-formyloxy-6,12-dihydroxy-abieta- 8,12-diene-11,14-dione (**111**), 7α-acetoxy-6,12-dihydroxy-abieta-8,12- diene-11,14-dione (**112**), 11-hydroxy-2α-(4-hydroxybenzoyloxy)- abieta-5,7,9(11),13-tetraene-12-one (**113**), 11-hydroxy-2α-(3,4-dihydroxybenzoyloxy) abieta-5,7,9(11),13-tetraene-12-one (**114**), 11-hydroxy-19-(methyl-buten-2-oyloxy)-abieta -5,7,9 (11),13-tetraene-12-one (**115**), 11-hydroxy-19-(4-hydroxy-benzoyloxy)-abieta-5,7,9(11),13-tetraene-12-one (**116**) and 11-hydroxy-19-(3,4-dihydroxybenzoyloxy)-abieta-5,7,9(11),13-tetraene-12-one (**117**). These compounds were tested for their anti-plasmodial activity and for their ability to inhibit β-haematin formation. Overall, they showed good activity (IC_50_ values ranging from 3.11 to 14.65 μM), with compound **114** being 62% as effective as chloroquine in inhibiting β-haematin formation. Compounds **111**, **114** and **117** were more active than quinine. However, the cytotoxicity profile indicated a low degree of specificity towards the malaria parasite. When combined with quinine, three compounds (**114**, **115** and **117**) interacted in an additive manner whereas compound **111** interacted synergistically [95].

Two other abietane diterpenes with anti-plasmodial activities; ferruginol (**118**) and 3-*O*-benzoylhosloppone (**119**), were respectively isolated by Koch *et al*. [96] and Achenbach *et al*. [97]. Compound **118** was isolated from the methanol extract of dried aerial parts (leaves and stems) of *Fuerstia africana* (Lamiaceae), a low-growing herb endemic to tropical East Africa [96], while compound **119** was isolated from the root bark of *Hoslundia opposita* (Lamiaceae), harvested in Tanzania [97]. The anti-malarial activity of compound **118**, determined using the D6 (chloroquine-sensitive derived from CDC Sierra Leone) clone of *P. falciparum*, showed a strong activity with an IC_50_ of 1.95 μg mL^-1^ compared to chloroquine IC_50_ = 1.94 μg mL^-1^[96]. Meanwhile, 3-*O*-benzoylhosloppone (**119**) inhibited the growth of the multidrug resistant strain K1 of *P. falciparum in vitro* with an IC_50_ value of 0.4 μg mL^-1^[97]. A bioactivity-guided fractionation of the petroleum ether extract of the leaves of *Hyptis suaveolens*, from Nigeria, led to the isolation of the abietane-type diterpenoid endoperoxide, 13α-*epi*-dioxiabiet-8(14)-en-18-ol (**120**), a molecule with high anti-plasmodial activity (IC_50_ = 0.1 μg mL^-1^) [98].

### Sesquiterpenes and sequiterpene lactones

Sequiterpenes derived from *Vernonia* sp. are known to have interesting anti-plasmodial activities. The compounds include vernodalin (**121**), vernodalol (**122**), vernolide (**123**), hydroxyvernolide (**124**), derived from the leaves of *Vernonia amygdalina* by Ohigashi *et al*. [99], in addition to 16,17- dihydrobrachycalyxolide (**125**) isolated from the leaves of the sister species, *Vernonia brachycalyx*, as a major anti-plasmodial compound, by Oketch-Rabah *et al*., Figures 15 and 16[100]. These compounds exhibited moderate anti-plasmodial activity against the multidrug-resistant K-1 strain of *P. falciparum*, vernodalin (**121**) being the most active compound with an IC_50_ value of 4 μg mL^-1^. Meanwhile, compounds **122**, **123** and **124** had IC_50_ values of 4.2, 8.4 and 11.4 μg mL^-1^, respectively [99]. The measured activities of the compounds correlates with the uses of the plants in ATM (the leaves of *Vernonia amygdalina* are used in the treatment of various diseases, including malaria). Quantitative analysis showed that young leaves of this species have a higher concentration of compound **121** than the other derived compounds, suggesting that the anti-malarial efficacy of the leaf extracts of this species may be partly due to the high content of this NP. It has also been reported that dry leaves of *Vernonia brachycalyx* contain 0.2-0.4% of the sesquiterpene dilactone **125**. This compound exhibited moderate to high anti-plasmodial activity against the K39, 3D7, V1/S and Dd2 *P. falciparum* strains, with IC_50_ values of 4.2, 13.7, 3.0, and 16 μg mL^-1^, respectively [100]. In spite of the anti-plasmodial activity of this compound, it also had higher toxicity against human lymphocytes, indicating that the anti-plasmodial activity may have been due to the general toxicity the compound had on cells. Despite these observations, the leaves of this species are still used in the treatment of malaria and parasitic infections in East Africa [45]. Ajugarin-1 (**126**) is another sesquiterpene, which has been reported from aerial parts of *Ajuga remota*, harvested in Kenya [101]. The compound has exhibited moderate anti-malarial properties against the chloroquine-sensitive FCA20/GHA strain of *P. falciparum*, with an IC_50_ of 23 μM [101].

The sesquiterpenoids oplodiol (**127**), 5*E*,10(14)-germacradien-1β,4β-diol (**128**) and 1(10) *E*,5*E*-germacradien-4α-ol (**129**), derived from *Reneilmia cincinnata*, with respective IC_50_ values of 4.17, 1.63 and 1.54 μM, were used to validate the use of this plant in ATM to cure malaria and other fevers in Cameroon [102]. In addition, Ganfon *et al*. investigated the antiparasitic activities of two sesquiterpenic lactones isolated from *Acanthospermum hispidum* harvested in Benin Republic [103]. From their results, two known sesquiterpenic lactones were isolated: 15-acetoxy-8β-[(2-methylbutyryloxy)]-14-oxo-4,5-*cis*-acanthospermolide), **130** and 9α-acetoxy-15-hydroxy-8β-(2-methylbutyryloxy)-14-oxo- 4,5-*trans*-acanthospermolide), **131**. Compounds **130** and **131** showed *in vitro* anti-plasmodial activity against the chloroquine-sensitive strain (3D7) with IC_50_ values of 2.9 and 2.23 μM, respectively. Only **131** showed a high selectivity index (SI: 18.4) on *Plasmodium* compared to cytotoxicity against human fibroblasts cell line (WI38). Furthermore, the crude acidic water extract and fractions containing one of the two isolated compounds displayed a weak *in vivo* anti-malarial activity against *P. berghei berghei* with a long half-life causing a delayed effect. *In vivo* acute (2000 mg kg^-1^) and sub-acute (1000 mg kg^-1^) toxicity tests of the crude acidic water extract did not show toxicity. Moreover, the crude acidic water extract, fractions and pure isolated compounds from *Acanthospermum hispidum* showed promising *in vitro* anti-plasmodial activity. Despite the fact that this study did not show *in vivo* acute and subacute toxicities of the crude acidic water extract, its weak *in vivo* anti-malarial activity and the *in vitro* cytotoxicity of pure compounds and enriched extracts containing **130** and **131** indicate that the aerial parts of this plant should be used with caution for malaria treatments [103].

The combined use of bioassay-guided fractionation based on *in vitro* anti-plasmodial assay and dereplication based on HPLC–PDA–MS–SPE–NMR by Pederson *et al*. [104], led to isolation of (6*S*,7*R*,8*S*)-14-acetoxy-8-[2-hydroxymethylacrylat]-15-helianga-1(10),4,11(13)-trien-15-al-6,12-olid or vernangulide A (**132**) and (5*R*,6*R*,7*R*,8*S*,10*S*)-14-acetoxy-8-[2-hydroxymethylacrylat]-elema-1,3,11(13)-trien-15-al-6,12-olid or vernangulide B (**133**), along with vernodalol (**134**), vernodalin (**135**) and 11,13β-dihydroxyvernodalin (**136**) from the dichloromethane/methanol 1:1 and methanol extracts of the aerial parts of *Distephanus angulifolius.* The isolated compounds showed IC_50_ values in the range 1.6 to 3.8 μM and 2.1 to 4.9 μM against chloroquine-sensitive D10 and chloroquine-resistant W2 *P. falciparum* strains, respectively. Janson *et al*. identified urospermal A-15-O-acetate (**136**) as the main active compound responsible for the anti-plasmodial activity of *Dicoma tomentosa* (Asteraceae) from Burkina Faso [105]. Based on their results, the IC_50_ of the compound was <1 μg mL^-1^ against both 3D7 and W2 strains. Compound **136** was found to be the main cytotoxic compound (SI = 3.3). A rapid quantification of the anti-malarial drug, artemisinin (**137**) in *Artemisia annua* plants cultivated for the first time in Burundi by Reale *et al*., revealed the prospect of cultivating *Artemisia* and eventually using the active principle to offer the population of Burundi a fundamental resource in a country where malaria is endemic [106].

Standard phytochemical analysis techniques, including solvent-solvent extraction, thin-layer- and column chromatography, were used by Becker *et al*. to isolate a eudesmanolide-type sesquiterpene lactone, dehydrobrachylaenolide (**138**), as the main active constituent of *Dicoma anomala* subsp. *gerrardii* from the Brits region of North West Province of South Africa [107]. The compound demonstrated an *in vitro* IC_50_ of 1.865 μM against a chloroquine-sensitive strain (D10) of *P. falciparum*. The biological activities of synthetic analogues of compound **138** showed that a methylene lactone group must be present in the eudesmanolide before any significant anti-malarial activity could be observed. This feature is absent in the artemisinins and suggests that eudesmanolide-type sesquiterpene lactones have a different mode of action from artemisinins. This hypothesis was further confirmed by microarray gene ontology analysis [107]. The ether extract from aerial parts of *Tithonia diversifolia* collected in São Tomé and Príncipe demonstrated good anti-plasmodial activity (IC_50_ of 0.75 μg mL^-1^ against the FCA strain) and fractionation of this extract yielded the sesquiterpene lactone tagitinin C (**139**) as an active compound against *P. falciparum* (IC_50_ of 0.33 μg mL^-1^ against the FCA strain) [108].

Okundoperoxide (**140**), a new compound with a cyclic endoperoxide moiety, was isolated by Efange *et al*. from *Scleria striatinux* (Cyperaceae), a spice commonly used in Cameroonian folk medicine to treat malaria and other fevers. This molecule exhibited significant anti-plasmodial activity, with IC_50_ values of 0.47, 0.48, 1.49, and 1.30 μg mL^-1^, on *P. falciparum* W2, D6, K1, and NF54, respectively. Moreover, the molecule showed no significant toxicity against mammalian cells [109].

### Coloratane sesquiterpenes

Wube *et al*. demonstrated the antiprotozoal activity of *Warburgia ugandensis* (Canellaceae) from Ethiopia towards *Trypanosoma brucei rhodesiense* and *P. falciparum in vitro* and attributed the anti-plasmodial activity to the presence of drimane and coloratane sesquiterpenes. These include 4(13),7-coloratadiene-12,11-olide (**141**), 11α-hydroxymuzigadiolide (**142**), muzigadial (**143**), 6α,9α-dihydroxy-4(13),7-coloratadiene-11,12-dial (**144**), cinnamolide (**145**), cinnamolide-3β-acetate (**146**), mukaadial (**147**) and ugandensidial (**148**), Figure 17. The anti-plasmodial assays also revealed that the six coloratane and six drimane sesquiterpenes isolated from this extract exhibited significant antitrypanosomal activity with IC_50_ values ranging from 0.45 to >114 μM. Among the compounds tested against the malaria parasite *P. falciparum* 11α-hydroxymuzigadiolide (**142**) was most active with an IC_50_ value of 6.40 μM [110].

### Beilshmiedic acid derivatives

Beilschmiedic acid derivatives exhibiting antibacterial and anti-plasmodial activities were obtained from *Beilschmiedia cryptocaryoides* (Lauraceae) collected from Madagascar (Table 5). The work of Talontsi *et al*. [111] led to the isolation of four new beilschmiedic acid derivatives, cryptobeilic acids A - D (**149** to **152**), and tsangibeilin B (**153**), Figure 19. Compounds **149** to **153** exhibited anti-plasmodial activity against erythrocytic stages of chloroquine-resistant *P. falciparum* strain NF54 (with IC_50_ values ranging from 5.35 to 17.70 μM) and weak cytotoxicity against L6 cell lines (with IC_50_ values ranging from 20.4 to 61.0 μM), the most promising antiplasmodial activity being shown by compound **150**.

### Pentacyclic triterpenes

The crude organic (methanol/dichloromethane (1:1)) extract of the leaves of *Schefflera umbellifera* (Araliaceae) exhibits promising anti-malarial activity. Bioassay-guided fractionation of this extract yielded the active compound, 3-hydroxy-20(29)-lupen-28-ol (**154**), Figure 19, which exhibited good anti-plasmodial activity (IC_50_ of 3.2 μg mL^-1^), when tested against a chloroquine-susceptible malarial strain (D10). The reference compound (chloroquine) gave an IC_50_ of 27.2 ng mL^-1^[112]. The quinonemethide triterpene, pristimerin or (20α)-3-hydroxy-2-oxo-24-nor-friedela-1(10),3,5,7-tetraen-carboxylic acid-(29)-methylester (**155**) was obtained by a bioactivity directed fractionation of the chloroform extract of the root bark of *Maytenus senegalensis* (Celasterceae) harvested from Sudan by Khalid *et al*. [113]. The *in vitro* anti-plasmodial activity of the isolated compound against chloroquine-resistant strain (Dd2) of *P. falciparum* was IC_50_ = 0.5 μg mL^-1^, while the cytotoxicity on lymphocyte proliferation model was detected at IC_50_ = 6.8 μg mL^-1^. The lupane-type triterpenoids 3-oxolupenal (3-oxolup-20(29)-en-30-al) (**156**), 3β-hydroxylupenal (3β-hydroxylup-20(29)-en-30-al) (**157**) and 3-oxolupenol (30-hydroxylup-20(29)-en-3-one) (**158**) were obtained from the leaves of *Nuxia sphaerocephala* (Loganiaceae), along with oleanolic acid (**160**), its acetylated ester (**159**), lupeol, uvaol, ursolic acid, and 3β-acetylursolic acid [83]. Among the compounds isolated from this study, **156** and **157** showed the best inhibitory activity against *P. falciparum* with the IC_50_ values between 1.55 and 4.67 μg mL^-1^*in vitro*, respectively.

Another lupane-type triterpene, lupeyl docosanoate (**162**), was isolated from the bark extract of *Hymenocardia acida* (Phyllanthaceae) collected in Chad, along with lupeol (**161**) and β-sitosterol by Mahmout *et al*. [114]. The anti-malarial property of compound **162** justifies the ethnobotanic use of the plant in the treatment of malaria. *Cassia siamea* (Fabaceae) was identified from an ethnobotanical survey of southwest Nigeria as a remedy for febrile illness. Bioassay-guided fractionation of stem bark of the plant extract, using the parasite lactate dehydrogenase assay and multi-resistant strain of *P. falciparum* (K1) for assessing the *in vitro* anti-malarial activity led to the isolation of emodin and lupeol (**161**) from the ethyl acetate extract [115]. Both compounds were found to be the active principles responsible for the anti-plasmodial property with IC_50_ values of 5 μg mL^1^, for each compound. The compounds 22-hydroxyhopan-3-one (**163**) and 24-methylene cycloartenol (**164**) from the stem bark of *Entandrophragma angolense* (Meliaceae) had moderate activities against *P. falciparum* W2 [90]. Zofou *et al*. evaluated the anti-plasmodial activity of betulinic acid (**165**) from the stem bark of the African St John’s wort, *Hypericum lanceolatum* (Hypericaceae). The compound had an IC_50_ of 2.05 μg mL^-1^[116]. The *n*-hexane extract of *Psorospermum glaberrimum* from Cameroon showed good anti-plasmodial activity against the *P. falciparum* W2 strain, with IC_50_ of 0.87 μg mL^-1^[117]. Lenta *et al*. isolated betulinic acid (**165**) and friedelan-3-ol (**166**) from this extract. The measured *in vitro* activity of compound **165** against the *P. falciparum* W2 strain gave an IC_50_ of 2.33 μg mL^-1^. Mbah *et al.* isolated 3-*O*-betulinic acid *p*-coumarate (**167**) from *Baillonella toxisperma*, with an IC_50_ of 1.65 μM [118]. The triterpenoid 2β,3β,19α-trihydroxy-urs-12-20-en-28-oic acid (**168**) was isolated by Zofou *et al*. [119] from the stem bark of *Kigelia africana* (Bignoniaceae). This compound exhibited an IC_50_ of 0.90 μg mL^-1^ against the W2 strain of *P. falciparum*.

*Cogniauxia podolaena* (Cucurbitaceae) is traditionally used in Congo Brazzaville for the treatment of malaria. The anti-plasmodial activity of the plant and some of the isolated compounds responsible for its activity were assessed by Banzouzi *et al*. [120]. Cucurbitacin B (**169**), cucurbitacin D (**170**) and 20-*epi*bryonolic acid (**171**) were assayed for anti-plasmodial activity (on FcM29, a chloroquine-resistant strain of *P. falciparum*) and cytotoxicity (on KB and Vero cell lines). The compounds showed respective IC_50_ values of 1.6, 4.0 and 2.0 μg mL^-1^ on FcM29. Compounds **169** and **170** both showed high cytotoxicity whereas **171** showed a better selectivity index.

## Conclusions

In this review an attempt has been made to document anti-malarial activities of NPs derived from African medicinal plants. It covers results published until the time of submission of the article. The first part of the review involves naturally occurring, anti-plasmodial/anti-malarial alkaloids and terpenoids while the second part of the review focuses on the remaining classes of compounds. Some of the compounds have been isolated from plants reputed to have a long history of usage in ATM, inferring that knowledge from ATM could be very useful in drug discovery efforts from African medicinal plants. From every indication, recent research efforts on new anti-malarial agents should focus on two main areas: the search for new chemical entities (NCEs) of natural/semisynthetic origin, and the development of phytomedicines [37]. It should be mentioned that African researchers have, knowingly or unknowingly, blown the former avenue out of proportion. This is basically as a result of the fact that most of the research activities on medicinal plants going on in Africa are carried out by academic research groups and the focus is on publications, not application. This calls for the need to develop the necessary applications required to turn acquired knowledge on NPs derived from African medicinal plants into concrete applications in phytomedicine, within an industrial setting. It has been noticed that among the anti-malarials mentioned in this review, most have never been tested for cytotoxicity and very few have been tested for *in vivo* antoplasmodial activity. Another limitation is the, often small, quantities of compounds isolated from the plants which frustrate ambitions of large-scale screening efforts. Since some complex anti-malarial mixtures derived from plant extracts sometimes loose their anti-malarial properties when pure compounds are isolated, due to synergism of molecules in mixture, the trend towards the development of total extracts into phytomedicines or improved traditional preparations is to be encouraged. Moreover, the isolation and characterization of NPs is an expensive endeavour, not within the reach of the average African research group. However, the attempt to validate ATM remedies as drugs will also face a number of limitations, among which are dosage determinations, variations of the concentration of the active ingredients in the plants with seasonal variations, the rapid loss of tropical forests and the extinction of key species, intellectual property rights management, the intervariability of plant species, quality control, and the conservation of biodiversity. The reconciliation between academic-oriented research and the development of phytomedicines could be feasible with the establishment of African centres of excellence in drug discovery [121], an initiative of the African Network for Drugs and Diagnostics Innovation (ANDI) [122], ATM being a major hub in this endeavour. In order to enhance modern drug discovery efforts from phytochemicals derived from the African flora, a recent effort by the authors of this paper has been to develop virtual libraries including NPs derived from African medicinal plants that have been reported in the literature, for computer-aided drug discovery (CADD). These include the CamMedNP database, containing three-dimensional structures of NPs derived from Cameroonian medicinal plants [123], the ConMedNP database, which covers ten countries in the Central African geographical region, converging the Congo Basin [124] and the AfroDb database, which is a select highly potent dataset, covering compounds with remarkable activities derived from plants across the entire continent [125]. Such databases could serve as starting points for virtual screening (VS) and CADD, leading to the identification of *in silico* hits, followed by validation by biological assays. These efforts have been in line with the prediction of DMPK profiles of the NPs, with a view to prioritizing hit selection during VS campaigns [125-127].

## Abbreviations

AfroDb: African medicinal plants active compound database; ATM: African traditional medicine; ADME/T: Absorption, distribution, metabolism, excretion, and toxicology; ANDI: African network for drugs and diagnostics innovation; CADD: Computer-aided drug design; CamMedNP: Cameroonian medicinal plant and natural products database; ConMedNP: Congo basin medicinal plant and natural products database; DMPK: Drug metabolism and pharmacokinetics; NP: Natural product; VS: Virtual screening; WHO: World Health Organization.

## Competing interests

The authors declare that they have no competing interests.

## Authors’ contributions

FNK, LLL, JCN, and LMM conceived the idea. FNK, LLL and PAO participated in the data collection. FNK and PAO contributed in the data analysis, the discussion of results and the conception of the paper under the supervision of LMM, WS, LLL, and JCN. FNK and PAO wrote the first draft of the paper and all authors agreed on the final version before submission.
